# Everyday Walking Among Older Adults and the Neighborhood Built Environment: A Comparison Between Two Cities in North America

**DOI:** 10.3389/fpubh.2020.564533

**Published:** 2020-12-22

**Authors:** Florian Herbolsheimer, Atiya Mahmood, Yvonne L. Michael, Habib Chaudhury

**Affiliations:** ^1^Department of Gerontology, Simon Fraser University, Vancouver, BC, Canada; ^2^Dornsife School of Public Health, Drexel University, Philadelphia, PA, United States

**Keywords:** physical activity, built environment, walking, neighborhood, older adults

## Abstract

A walkable neighborhood becomes particularly important for older adults for whom physical activity and active transportation are critical for healthy aging-in-place. For many older adults, regular walking takes place in the neighborhood and is the primary mode of mobility. This study took place in eight neighborhoods in Metro Portland (USA) and Metro Vancouver (Canada), examining older adults' walking behavior and neighborhood built environmental features. Older adults reported walking for recreation and transport in a cross-sectional telephone survey. Information on physical activity was combined with audits of 355 street segments using the Senior Walking Environmental Audit Tool-Revised (SWEAT-R). Multi-level regression models examined the relationship between built environmental characteristics and walking for transport or recreation. Older adults [*N* = 434, mean age: 71.6 (*SD* = 8.1)] walked more for transport in high-density neighborhoods and in Metro Vancouver compared to Metro Portland (*M* = 12.8 vs. *M* = 2.2 min/day; *p* < 0.001). No relationship was found between population density and walking for recreation. Older adults spent more time walking for transport if pedestrian crossing were present (*p* = 0.037) and if parks or outdoor fitness amenities were available (*p* = 0.022). The immediate neighborhood built environment supports walking for transport in older adults. Comparing two similar metropolitan areas highlighted that high population density is necessary, yet not a sufficient condition for walking in the neighborhood.

## Introduction

The literature widely supports the health benefits for older adults who engage in regular physical activity [e.g., (1, 2)]. Thereby, walking is the most popular form of physical activity among older adults (3, 4). The neighborhood built environment plays a significant role in walking for recreation or transport, which generally takes place outdoors and in nearby settings (e.g., parks, shopping malls, trails, neighborhood streets) (5).

As postulated by social-ecological models, walking is affected by multiple levels of influence, including the built environment (6, 7). Therefore, walking needs to be analyzed from a multi-level perspective bringing together individual characteristics and physical environmental features. Older adults with declined functioning are more likely to be more affected than other age groups by the neighborhood built environmental features as being supportive or restrictive (8).

It has been suggested that older adults walk more often in high-dense residential areas (9). Residential density and the walkability of the built environment, such as easy access to destinations and services, pedestrian-oriented street elements and street patterns, and connectivity, are associated with physical activity and walking (10). By distinguishing different types of walking behavior, a review revealed strong relationships between walking for transport and walkability, urbanization, land use mix, accessibility, and the presence of amenities (11). Walking for recreation was closely associated with walkability, aesthetics/green spaces, and air quality.

However, most research on the built environment and walking has not fully accounted for the wide variation in built-environmental characteristics. Most studies relied on single-countries, primarily the U.S. and other high-income countries with limited variability in density resulting in an underestimation of potential effects. As a result, reviews of the neighborhood built environment and older adult's walking activity reveal inconsistent findings (11). Studies adopting a similar research design in Japan and Taiwan have found no association between public transport and walking for transport in older adults (12, 13), whereas public transport was positively related to walking for transport in Belgium, the U.S., and China (14–16). Other studies reported that walkable neighborhoods, notably higher density environments, were associated with walking for transport in older adults (15, 17, 18), but not in other studies (13, 19, 20).

Therefore, an international comparison approach is crucial because unique built environmental conditions related to issues such as local topography, urban planning, social preferences, etc. are likely to modify the relationship between walking and the neighborhood built environment. An exception is the International Physical Activity and the Environment Network (IPEN) study across 11 countries. Land-use mix and sidewalks showed the most consistent associations with moderate-to-vigorous physical activity (21). However, single study sites are hard to compare because they differ in population densities. The findings of the IPEN study can only partially be transferred to an older adult population as it represents a younger population with a mean age of forty-two.

The present study applies a country-comparative perspective analyzing the neighborhood-built environment and walking in older adults. Districts in both counties were selected based on the population's density and income. Combining a survey and a street-level built environmental audit, we examined walking behavior of older adults in terms of: (1) the differences between the U.S. and Canadian metropolitan areas, (2) its associations with population density, and (3) its associations with neighborhood built environmental factors within and across study sites.

## Materials and Methods

This study was part of a larger three-phase, mixed-methods research project consisting of a qualitative photovoice method (22) a neighborhood environmental audit using the Senior Walking Environmental Audit Tool-Revised (SWEAT-R) (23, 24), and a telephone survey (25). The study was conducted in the metropolitan areas of Vancouver, British Columbia (B.C.), Canada and Portland, Oregon (OR), United States. The topography, climate, and urban planning decisions had broad similarities due to the two cities' locations in North America's Pacific Northwest. Given these similarities, we wanted to explore if physical activity was also similar among the older adult populations in comparable built environments. Data collection took place in four neighborhoods in the Metro Vancouver and four neighborhoods in Metro Portland. Census tract data were used to select eight neighborhoods based on neighborhood density and income levels to ensure variation in the physical environment features essential for physical activity (23). The following analyses utilize a cross-sectional telephone survey conducted in a random sample of older adults from the eight neighborhoods and combine this information with street audits of the corresponding neighborhoods. The study was approved by the Simon Fraser University Ethics review committee (Number 38156).

### Study Population

A detailed description of the study is published elsewhere (25). In short: 434 older adults completed the telephone survey, among whom 393 reported being physically active in their neighborhood or outdoor places. Eligibility criteria for participants were: (a) at least 60 years of age at the time of the survey, (b) living in one of the selected neighborhoods, and (c) being able to understand English. These older adults resided across eight neighborhoods: Mount Tabor, OR (*n* = 56), Clackamas, OR (*n* = 50), Lake Oswego, OR (*n* = 61), Milwaukie, OR (*n* = 64), Vancouver, BC (*n* = 53), Burnaby, BC (*n* = 51), South Surrey, BC (*n* = 50) and Maple Ridge, BC (*n* = 49).

## Measures

### Walking

Recreational walking and walking for transport was operationalized in two ways in the following analyses: (1) as a dichotomous outcome (currently physically active or not), and (2) time spent per day for a specific activity was operationalized as a continuous measure. To assesses whether or not participants were physically active, we asked if the participants engaged in a list of physical activities (i.e., gardening, housework, walking for transport, walking for recreation, etc.) in the previous 4 weeks (yes/no). In a second question, the participants provided information on the type and the frequency of up to three most common physical activity (or activities) they have engaged every week (metric score). The frequency and duration were multiplied and divided by seven days to calculate the average daily walking activity (in minutes/day). The following analyses only applied walking for recreation (e.g., walk in the park) and walking for transport (e.g., walking to a bank or grocery store). Extreme outliers (>4 SD) were identified and set to the value of the 4th standard deviation (*n*_recreation_ = 2; *n*_transport_ = 4). Given the absence of an objective, or a gold standard measure of walking, we evaluated both walking measures (i.e., metric walking measure in minutes per day and dichotomous walking measure in yes/no) in our analyses to compare them and evaluate the robustness of the results based on differently operationalized walking activities.

### Seniors Walking Environment Assessment Tool-Revised (SWEAT-R)

The SWEAT-R was developed as a tool to collect data on the physical environment to understand its association with physical activity. It is organized into four domains, which are (a) functionality, (b) safety, (c) aesthetics, and (d) destinations, and has been shown a valid tool with high inter-rater reliability (23, 24). In the current study, SWEAT-R data were collected along with a sample of street segments (defined as the street section between two intersections) in each neighborhood, excluding highways. Correlates of walking, such as street connectivity and land-use, were addressed by including low and high residential density neighborhoods in this study. These segments in the eight neighborhoods were randomly selected, with a range from 16 to a maximum of 58 audited segments per neighborhood. In total, 158 segments were observed across the four Portland metropolitan neighborhoods, and 197 segments were audited across the four Vancouver metropolitan neighborhoods.

Four research assistants (two per region) received training before data collection. Training led by research investigators encompassed both classroom and field components. Training manuals with detailed explanations for each item in SWEAT-R were provided, and the second observation form was discussed in detail. For each street segment, the research assistant collected data on 168 environmental characteristics. If items were present in fewer than 2 percent of all segments, they were deleted from subsequent analyses (*n* = 63). Factor scores were calculated for each of the four dimensions based on the remaining items and resulted in 35 factors. Five factors were excluded from the following analyses as they did not vary significantly across the eight districts. To identify the most relevant factors, we calculated correlation analyses with Bonferroni-adjusted significance levels between walking and the SWEAT-R extracted factors and accepted factors that reached a significance level of *p* < 0.001. Finally, 10 factors remained significant in the correlation analyses, of which four reached significance in the multi-level models.

### Covariates

Background characteristics included sex, age, self-rated health, the average of the maximum temperature in the last 4 weeks (in degrees Celsius), duration of residency (in years), and country of residence were considered as potential confounders that have been used in previous studies (12, 13, 26). Self-rated health was measured with one item from the 12-item Short-Form Health Survey: “In general would you say your health is” including poor, fair, good, very good, and excellent (27). The duration of residence was assessed by asking, “How many years have you lived in your current neighborhood?” Moving within the neighborhood counted to the total years. Local weather stations provided average values for temperature in degrees Celsius (°C) for each of the 28 days before the completion of the physical activity questionnaire. For each participant, the average temperature was calculated. Additionally, sex and age (in years) were assessed.

### Statistical Analysis

Data were analyzed using STATA 15.0 (StataCorp L.P., College Station, TX). Baseline demographic and health characteristics are presented stratified by country and the density of neighborhoods. Differences in mean were tested using a one-way analysis of variance (ANOVA), and frequencies were tested using the Pearson Chi-square Test. First, a linear regression was conducted to compare the effect of the neighborhood density and the location (the USA vs. Canada) and test for an interaction between country and population density. Second, using the Intraclass Correlation Coefficient (ICC) (variance due to neighborhood/total variance), the neighborhood accounted for 13% of the time spent walking for transport and 6% for recreational walking. That justified using a mixed modeling approach to analyze walking for transport, including neighborhood as a random effect (Vajargah and Nikbakht, 2015). Generalized linear mixed models with gamma log link transformation (with the value of one added to all scores to eliminate zeros) and multi-level logistic regression models were used to study associations between the neighborhood characteristics and walking activity. Each environmental attribute (based on the street audits) was analyzed in a separate model. All models were adjusted for age and sex, self-reported health, mean maximum temperature over the last 4 weeks, and the duration of residency in the neighborhood. Likert-type variables with <5% item non-response were imputed using mean values from valid records.

## Results

The characteristics of the study sample are presented stratified by density and country in Table 1. Respondents (*N* = 434) were, on average 71.6 (*SD* = 8.1) years old, mostly female (64.7%), highly educated (44.0%), and had an average total annual household income of $47,300 (*SD* = 22,300). Overall, the participants walked more often for recreation than for transport (74.2 vs. 49.3%) in the previous 4 weeks, which is reflected by an average of 5.4 min per day (*SD* = 12.2) for transport and 13.4 min per day (*SD* = 16.5) for recreational purposes. Comparing districts in Metro Portland and Metro Vancouver with high density and low density, significant differences were found for education attainment, mean temperature, duration of residency in the neighborhood and both walking measures. *Post-hoc*-tests revealed that older adults walked significantly longer for recreation when comparing low-density neighborhoods between Metro Vancouver and Metro Portland (*M* = 20.0 vs. *M* = 7.6; *p* < 0.001). Walking for transport was significantly higher in the high-density neighborhoods of metro Vancouver compared to high-density neighborhoods in Portland (*M* = 12.8 vs. *M* = 2.2; *p* < 0.001).

**Table 1:** Sample characteristics of respondents, neighborhood and physical activity study (*n* = 434), Vancouver, British Columbia, Canada and Portland, Oregon, United States of America.

	**Overall**		**Vancouver**				**Portland**					
			**High density (*****n*** **=** **104)**		**Low density (*****n*** **=** **99)**		**High density (*****n*** **=** **120)**		**Low density (*****n*** **=** **111)**		**χ^2^/F**	***p*-value**
Age (years), M (SD)	71.6	(8.1)	72.0	(7.9)	71.1	(7.7)	70.8	(8.3)	72.4	(8.3)	1.0	0.387
Female, (%)		64.7	66.3		56.6		64.2		71.2		5.4	0.147
Married, (%)		52.3	47.1		63.6		52.5		46.7		7.5	0.056
Education, (%)												
High school or less		24.2	23.1		34.3		18.3		22.5			
Some post-secondary		31.8	22.1		30.3		37.5		36.0			
Completed college/university		44.0	54.8		35.4		44.2		41.5		15.7	0.015
Total annual household income (thousand $), M (SD)	47.3	(22.3)	46.7	(21.6)	49.4	(22.5)	51.2	(21.6)	43.0	(22.9)	2.2	0.084
Duration of residence (years), M (SD)	21.9	(15.4)	19.3	(12.6)	16.4	(12.8)	26.2	(16.1)	24.7	(17.2)	10.2	<0.001
Walking for transport, (%)		49.3	78.8		59.6		39.2		23.4		75.2	<0.001
Walking for recreation, (%)		74.2	77.9		77.8		75.0		66.7		4.7	0.193
Walking for transport^a^ (minutes/day), M (SD)	5.4	(12.2)	12.8	(18.0)	6.2	(11.9)	2.2	(7.0)	1.2	(4.7)	23.0	<0.001
Walking for recreation (minutes/day)^b^, M (SD)	13.4	(16.5)	15.6	(17.7)	20.0	(20.4)	11.3	(13.6)	7.6	(10.9)	12.1	<0.001
Mean temperature (°C), M (SD)	10.0	(3.0)	10.0	(4.3)	10.3	(2.6)	9.3	(1.2)	10.4	(3.0)	3.3	0.021
Self-rated health^c^, M (SD)	3.7	(1.0)	3.6	(0.9)	3.8	(1.0)	3.8	(1.0)	3.8	(1.0)	1.2	0.314

a *Ranges from 0 to 63.4*.

b *Ranges from 0 to 81.7*.

c *Possible range from 1 to 5, higher values indicate better health*.

**Figure 1:**
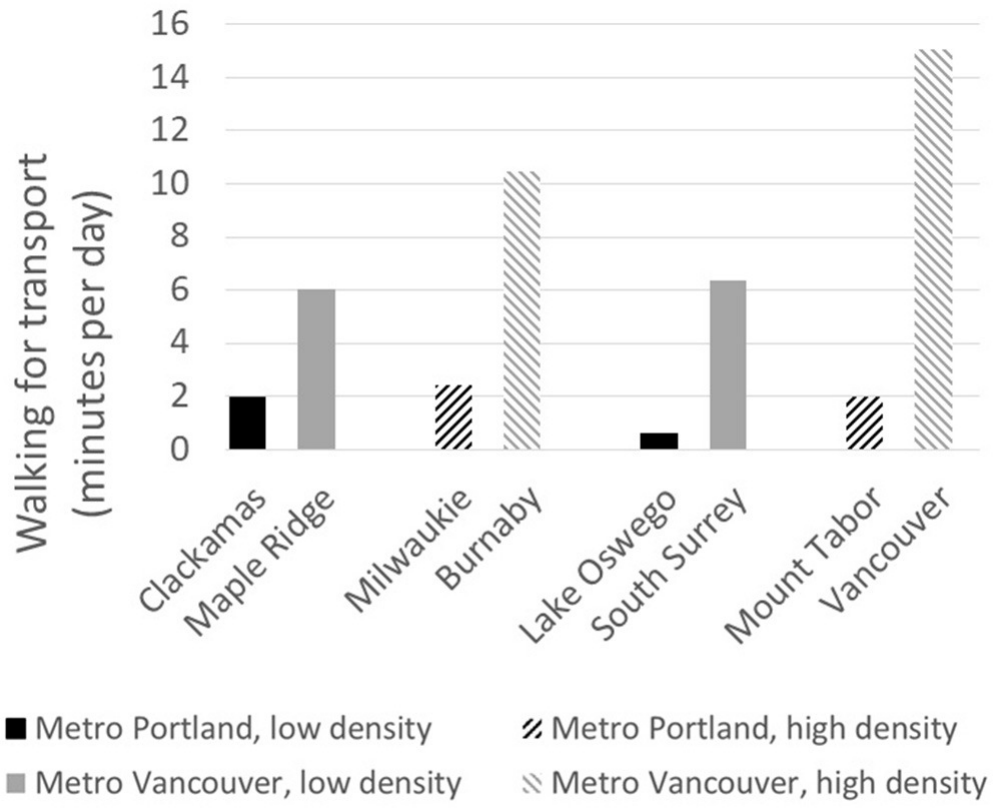
Mean walking for transport time in eight neighborhoods.

Figure 1 illustrates these variations in walking for transport across the eight neighborhoods of our study. Walking for transport ranged from 0.6 min per day in Lake Oswego to 15.1 min in the Vancouver neighborhood. It showed that Canadians walked more often for transport (Maple Ridge, Burnaby, South Surrey, and Vancouver neighborhood). Older adults also walked more for transport in high-density neighborhoods (Milwaukie, Burnaby, Mount Tabor, and Vancouver neighborhood) across the two metro areas. Accordingly, the linear regression showed that older adults in Metro Vancouver walked on average 10.1 more min per day for transport compared to those in Metro Portland (Table 2). Overall, there was also more walking for transport in high-density neighborhoods compared to the low-density neighborhood. The interaction between density and country reached significance (*p* = 0.008), which means that older adults walked most for transport in high-density Canadian neighborhoods.

**Table 2:** Country of residence, population density, and walking activity (*n* = 393).

	**Walking for transport**		**Recreational walking**	
	***B***	***p*-value**	***B***	***p*-value**
**Main effects**				
USA (Ref. Canada)	−10.1	<0.001	−5.6	0.065
Low-dense district (Ref. High-dense)	−7.0	<0.001	4.1	0.065
**Interactions effect**				
Low-dense^*^ USA	5.9	0.008	−7.1	0.020

*Adjusted for age, sex, self-rated health, average maximum temperature, duration of residency*.

* *stands for the interaction terms which is the multiplication of both variables*.

### Descriptive Results From SWEAT-R Observations in Districts With a High Population Density

Descriptive audit data revealed an additional source of information about the variations between the high-density districts in Metro Vancouver (Burnaby, Vancouver) and Metro Portland (Milwaukie, Mount Tabor). Descriptive results summarized in Table 3 are based on a subset of SWEAT-R items that exhibited the highest degree of difference in observer responses. Each item score denotes the percentage of segments on which it was observed within a given neighborhood and the significance of percentage differences.

**Table 3:** Characteristics of street segments in high-density neighborhoods (*n* = 187 segments), Vancouver, British Columbia, Canada and Portland, Oregon, United States of America.

	**Burnaby**	**Milwaukie**			**Vancouver**	**Monut Tabor**		
	**(CA)**	**(USA)**			**(CA)**	**(USA)**		
**Units (% of segments)**	**(*n* = 56)**	**(*n* = 58)**	**χ^2^**	***p*-value**	**(*n* = 45)**	**(*n* = 28)**	**χ^2^**	***p*-value**
**Functionality**								
**Building types**								
Single-family homes (detached)	42.2	96.4	21.8	<0.001	21.4	96.6	66.8	<0.001
Low-rise multi-family housing (<5 stories)	37.8	14.3	4.6	0.031	30.4	5.2	12.5	0.001
High-rise multi-family housing (5 or more stories)	37.8	3.6	10.9	0.001	16.1	0.0	10.1	0.001
Grocery store	11.1	0.0	3.3	0.068	5.4	1.7	1.1	0.292
Pharmacy/drug store	6.7	0.0	1.9	0.163	1.8	0.0	1.0	0.307
Health clinics, medical facilities	13.3	3.6	1.9	0.168	3.6	1.7	0.4	0.538
Beauty/barber shop	6.7	0.0	1.9	0.163	5.4	0.0	3.2	0.074
Service facilities (e.g., insurance offices, dry cleaners)	13.3	0.0	4.1	0.044	17.9	0.0	11.4	0.001
**Sidewalks**								
Presence of sidewalks	93.3	14.3	46.3	<0.001	100.0	89.7	6.1	0.013
Continuous sidewalks on both sides (if present)	71.1	0.0	35.5	<0.001	58.9	56.9	0.0	0.826
**Public spaces**								
Park/playground	15.6	0.0	4.8	0.028	17.9	10.3	1.3	0.248
Outdoor fitness/recreation area	33.3	7.1	6.6	0.010	35.7	10.3	10.4	0.001
**Safety and Comfort**								
Crossing area with ramps or curb cuts	91.1	25.0	33.5	<0.001	73.2	34.5	17.2	<0.001
Grooves or bumps	75.6	21.4	20.4	<0.001	58.9	10.3	29.9	<0.001
Intended crossing area for pedestrians	11.1	7.1	0.3	0.576	8.9	3.5	1.5	0.223
Signs for pedestrians/children/etc.	37.8	10.7	6.4	0.012	28.6	1.7	16.2	<0.001
Signs for school speed zone	8.9	3.6	0.8	0.382	4.4	3.5	0.1	0.813

*N refers to the number of audited street segments in each neighborhood*.

The building types differed between the two Metro Vancouver and the two Metro Portland neighborhoods with high population density: In Portland, single-family detached houses are more prevalent, while in Vancouver, multi-family housing is far more common. The SWEAT-R documented that commercial destinations like grocery stores, barber shops, health clinics, and pharmacies were rare in high-density Metro Portland neighborhoods with only 0.0–3.6% of the audited streets having these destinations. In comparison, these destinations were more often present in each of the Metro Vancouver neighborhoods (1.8–17.9%).

Differences in sidewalk availability were observed between the high-density neighborhoods of Metro Vancouver and Metro Portland. A higher proportion of segments with sidewalks, as well as continuous sidewalks on both sides of the street, were more often present in Vancouver neighborhoods compared to Portland neighborhoods. Parks and outdoor fitness venues were more present in the Vancouver neighborhoods in comparison to the neighborhoods in Portland. Parks were more often present in the Vancouver low-income district (Burnaby) when comparing it with the Portland low-income district (Milwaukie) (15.6 vs. 0.0; *p* = 0.028), and outdoor recreational areas were also found to be three times more often present in the Vancouver neighborhoods. Street conditions that either slowed down the car traffic (e.g., bumps or grooves) or enabled pedestrians to cross the street safely (e.g., indented crossing areas) were significantly more prevalent in Vancouver neighborhoods, with these conditions most common in Burnaby.

### Associations of Audited Environmental Attributes With Walking Activity

In the last step, multi-level models were applied to identify the association between built environmental characteristics (based on the audits) and walking for transport (based on the survey). The multi-level models identified four significant environmental factors (Table 4): Building types, the presence of public spaces (i.e., parks and outdoor fitness), brick sidewalks, and safety (i.e., pedestrian crossings) were significantly (all *p* ≤ 0.050) positively associated with walking for transport in the linear mixed-model. Applying a multi-level logistic regression model, two built neighborhood characteristics remained significant: building types (OR = 1.81; *p* = 0.034) and street crossing areas for pedestrians (OR = 5.15; *p* = 0.001). The results were consistent with a dominance analysis (28) identifying the safety from traffic aspects (street crossing, traffic calming, the safety of intersection) as the essential aspects for walking for transport and neighborhood density and the presence of undeveloped land as the least relevant (Supplementary Material). None of the environmental attributes was significantly associated with walking for recreation.

**Table 4:** Association of environmental attributes with walking for transport, neighborhood and physical activity study (*n* = 434), Vancouver, British Columbia, Canada and Portland, Oregon, United States of America.

	**Walking for transport**					
**Environmental attribute**	**Linear model^a^**			**Logit model**		
	**B**	**(95% CI)**	***p*-value**	**OR**	**(95% CI)**	***p*-value**
**Buildings**						
Mixed-use houses	−1.44	(−3.95, 1.08)	0.263	0.05	(0.00, 1.15)	0.061
Buildings types^b^	0.42	(0.03, 0.81)	0.037	1.81	(1.05, 3.13)	0.034
Undeveloped land	−0.66	(−1.26, −0.06)	0.031	0.50	(0.19, 1.30)	0.154
**Sidewalks**						
Brick sidewalks	0.54	(0.15, 0.93)	0.006	1.92	(0.95, 3.87)	0.068
**Public spaces**						
Benches	0.76	(−0.13, 1.64)	0.093	2.37	(0.64, 8.82)	0.198
Green open space^c^	0.88	(0.13, 1.63)	0.022	3.01	(0.97, 9.35)	0.057
**Safety from traffic**						
Intersection^d^	0.48	(−0.04, 1.0)	0.069	1.66	(0.75, 3.67)	0.212
Street crossing^e^	0.91	(0.06, 1.76)	0.037	5.15	(2.02, 13.15)	0.001
Traffic-calming^f^	0.93	(−0.17, 2.02)	0.098	3.14	(0.58, 16.92)	0.183

OR, odds ratio; B, unstandardized coefficient;

a Multilevel linear mixed model with gamma log transformation;

b Few single family house, low rise multi-family house, high-rise multi-family house

c parks, outdoor fitness

d ramps or curb cuts

e Intended crossing area for pedestrians, signs for pedestrians, signs for school speed zone;

f *sidewalk extension, median strip; All models were adjusted for respondents' age, sex, self-rated health, duration of residency, country of residence, and mean maximum temperature*.

## Discussion

The study is unique as it compares equivalent neighborhoods with comparable socioeconomic composition and population density in an international context. The study revealed that time spent walking for transport in the two North American metropolitan areas is highly variable as older adults walked more for transport in high-density districts in Metro Vancouver compared to individuals in high-density districts in Metro Portland. Population density served as one important prerequisite but did not necessarily lead to walking for transport. The interplay of high-density neighborhoods with safe street crossings and nearby nature might motivate older adults to choose walking for transport.

In line with the existing literature, strong evidence was found for residential density and parks and walking for transport within the neighborhood (29). Outdoor recreational facilities that are easy to access and located within walking distance from home have been found to promote physical activity of community-dwelling older adults (30). The finding for residential density is especially relevant, given current policy decisions in U.S. cities to increase density to address the shortage of housing (31). Given research supporting the importance of increased density for higher levels of physical activity across the lifespan, these policies are also consistent with improving health behaviors. The positive association between safety from traffic and walking for transport is consistent with an earlier meta-analysis and several studies (15, 32, 33). That points to the need to advocate for policy and regulatory actions aimed at improving pedestrian safety.

A recent review revealed no environmental characteristics associated with walking regardless of the walking type (e.g., total, for transport, for recreation) among older adults (11). Our study did not find any significant associations between neighborhood characteristics and walking for recreation. This is in line with previous research that neighborhood characteristics served as more critical predictors for walking for transport rather than recreational walking among older adults (11, 34, 35) and among the entire population (36). The weak association might reflect that walking for recreation often takes place outside the immediate neighborhood as living in less-walkable neighborhoods might make persons seek more desirable places outside their own neighborhood (37).

No relationship was found between the population income and walking for transport or recreational walking when comparing low and high-income districts. This in accordance with previous research that identified differences between low and high-income districts when it came to moderate-to-vigorous physical activity but found no effect on walking (38).

We found a weak association between sidewalk characteristics and walking. We had expected the pedestrian infrastructure- specifically sidewalks-to be an essential neighborhood feature because it was one out of five attributes in a review of qualitative studies related to physical activity in older adults (39). In the IPEN study, which comprises of a younger population, the presence of sidewalk was one of the most consistent associations with moderate-to-vigorous physical activity (21). In contrast, no association was observed between walking in older adults and the percentage of sidewalks in a randomized controlled trial in Portland (40). While sidewalks were rarely present in Portland, they were more often available in Vancouver, which, however, did not translate to more time spending for walking for transport.

In contrast to prior research, we did not find that older adults who live within walking distance of shopping areas and public transit were more physically active than individuals who lived further away (29, 41–43). Nearby destinations did not reach significance even though commercial destinations and shops were more frequent in the Canadian neighborhoods in comparison to the American neighborhoods. Comparing similar neighborhoods that are equivalent in terms of population density and income might have leveled-out differences in nearby destinations.

We observed different walking for transport patterns in our study, although the metropolitan areas of Vancouver and Portland are located in the same geographic region (Pacific Northwest of North America) with comparable cultural backgrounds and similar demographic characteristics (in terms of age, sex, health status, and physical limitations) and the same population density and income. Among other factors such as attitude to physical activity, built environmental differences might have resulted in diverse opportunity structures for walking in the neighborhood and made Canadians became even more physically active in 2005 compared to 1994 (44). In contrast, walking declined significantly in the United States among persons 65 and older while walking increased slightly in the general population (45).

This study has a few notable strengths in advancing our understanding of neighborhood influences on walking in older adults. Using data from two cities and eight neighborhoods matched for mean household income and population density, this study aimed to contribute to the understanding of how specific neighborhood environmental features are related to everyday walking levels. Single country and country-adjusted analyses cannot provide information on differences in country-level environments walking associations if population and neighborhood characteristics vary. Consequently, this study was also able to estimate the extent to which between-site differences explain differences in walking for transport patterns. The use of an environmental audit performed by two raters in several randomly chosen segments in each district made the study unique and less dependent on the participants' perception of the built environment. Studies have shown that physically active persons also tend to report more activity-friendly features and perceive their neighborhood to be more favorable in terms of physical activity supporting characteristics (46).

Although the study addresses a series of methodological issues, there are a few limitations. First, cross-sectional data limit the causal inferences about the neighborhood environment and walking behavior. Second, the study was conducted in a specific geographic region of North America, which might restrict the comparability to the two countries at large and other world regions. Another limitation was that the physical activity questionnaire only assessed up to the three most common activities, which might have led to an underestimate of activities. To address this issue, we presented results twofold: (1) as a metric measure based on the three most common physical activities using linear regression, and (2) as a dichotomous measure based on a different survey question using logistic regression. We demonstrated that the results were stable. Furthermore, we did not adjust for additional relevant confounding variables such as car ownership, race/ ethnicity, and residential self-selection. Last, walking measure was based on self-reports, which overestimate activity compared to objective measures and might be biased by other individual characteristics like cognitive function (47). However, these biases should work in each neighborhood in the same way and are less likely to affect the association between the built environment and walking behavior.

## Conclusions

Supporting walking in older adults is critical to the development of healthy cities. Public policies on urban infrastructure development shape individual transportation choices by setting the conditions of personal cost, benefits, and opportunities. A safe and pedestrian-friendly neighborhood might positively influence walking for transport behavior. This research indicates that it is essential to consider a combination of physical planning characteristics to foster everyday walking behavior in older adults effectively. There is a need for policy and regulatory actions that consider increasing housing density and other contributing factors, such as appropriately designed street crossing, the presence of parks, etc. Future research can further examine, among other topics, countries, and jurisdictions that have notably different city planning and urban design contexts and associated potential variability in walking behaviors in older adults.

## Data Availability Statement

We usually do not state this about the data because of the university ethics consideration. We prefer “Sharing of raw data would be contingent on approval from the university research ethics office.”

## Ethics Statement

The studies involving human participants were reviewed and approved by Simon Fraser University Office of Research Ethics. The patients/participants provided their written informed consent to participate in this study.

## Author Contributions

HC, AM, and YM contributed to the conception and design of the study. HC and FH organized the database. FH performed the statistical analysis and wrote the first draft of the manuscript. FH, AM, HC, and YM wrote sections of the manuscript. All authors contributed to manuscript revision, read, and approved the submitted version.

## Conflict of Interest

The authors declare that the research was conducted in the absence of any commercial or financial relationships that could be construed as a potential conflict of interest.
