# Supplementary material for: Everyday Walking Among Older Adults and the Neighborhood Built Environment: A Comparison Between Two Cities in North America
Source: Front Public Health. 2020 Dec 22;8:564533. doi: 10.3389/fpubh.2020.564533 (PMC7793713; doi:10.3389/fpubh.2020.564533)
Supplement: Supplementary file 1 [file Table_1.DOCX]

Supplement 1. Dominance analysis for hierarchical linear models with walking for transport as outcome

|  | Dominance analysis | |
| --- | --- | --- |
|  | Standardized dominance statistic | Rank |
| BUILDINGS |  |  |
| Mixed-use houses | 0.115 | 3 |
| Buildings types^a^ | 0.072 | 9 |
| Undeveloped land | 0.059 | 10 |
|  |  |  |
| SIDEWALKS |  |  |
| Brick sidewalks | 0.090 | 6 |
|  |  |  |
| PUBLIC SPACES |  |  |
| Benches | 0.075 | 8 |
| Green open space^b^ | 0.078 | 7 |
|  |  |  |
| SAFETY FROM TRAFFIC |  |  |
| Intersection^c^ | 0.122 | 2 |
| Street crossing^d^ | 0.104 | 5 |
| Traffic-calming^e^ | 0.128 | 1 |
|  |  |  |
| Neigborhood density | 0.045 | 11 |
| Country of residence | 0.113 | 4 |
| *Note.* Dominance analysis for hierarchical linear models; ^a^ few single family house, low rise multi-family house, high-rise multi-family house; ^b^ parks, outdoor fitness; ^c^ ramps or curb cuts; ^d^ Intended crossing area for pedestrians, signs for pedestrians, signs for school speed zone; ^e^ sidewalk extension, median strip | | |
